# FOXO Regulates Organ-Specific Phenotypic Plasticity In *Drosophila*

**DOI:** 10.1371/journal.pgen.1002373

**Published:** 2011-11-10

**Authors:** Hui Yuan Tang, Martha S. B. Smith-Caldas, Michael V. Driscoll, Samy Salhadar, Alexander W. Shingleton

**Affiliations:** 1 Department of Zoology, Michigan State University, East Lansing, Michigan, United States of America; 2 Division of Biology, Kansas State University, Manhattan, Kansas, United States of America; 3 BEACON Center for the Study of Evolution in Action, Michigan State University, East Lansing, Michigan, United States of America; Swiss Federal Institute of Technology Zurich, Switzerland

## Abstract

Phenotypic plasticity, the ability for a single genotype to generate different phenotypes in response to environmental conditions, is biologically ubiquitous, and yet almost nothing is known of the developmental mechanisms that regulate the extent of a plastic response. In particular, it is unclear why some traits or individuals are highly sensitive to an environmental variable while other traits or individuals are less so. Here we elucidate the developmental mechanisms that regulate the expression of a particularly important form of phenotypic plasticity: the effect of developmental nutrition on organ size. In all animals, developmental nutrition is signaled to growing organs via the insulin-signaling pathway. *Drosophila* organs differ in their size response to developmental nutrition and this reflects differences in organ-specific insulin-sensitivity. We show that this variation in insulin-sensitivity is regulated at the level of the forkhead transcription factor FOXO, a negative growth regulator that is activated when nutrition and insulin signaling are low. Individual organs appear to attenuate growth suppression in response to low nutrition through an organ-specific reduction in *FOXO* expression, thereby reducing their nutritional plasticity. We show that *FOXO* expression is necessary to maintain organ-specific differences in nutritional-plasticity and insulin-sensitivity, while organ-autonomous changes in *FOXO* expression are sufficient to autonomously alter an organ's nutritional-plasticity and insulin-sensitivity. These data identify a gene (FOXO) that modulates a plastic response through variation in its expression. FOXO is recognized as a key player in the response of size, immunity, and longevity to changes in developmental nutrition, stress, and oxygen levels. FOXO may therefore act as a more general regulator of plasticity. These data indicate that the extent of phenotypic plasticity may be modified by changes in the expression of genes involved in signaling environmental information to developmental processes.

## Author Summary

The ability of an organism to respond to its environment is a defining quality of life. However, why are some characteristics or individuals sensitive to environmental change while others are not? We identified the mechanism that controls the response of growing organs to a particularly important environmental factor—developmental nutrition. In all animals, a decrease in developmental nutrition reduces final body and organ size. However, the size of some organs is less responsive to changes in nutrition than others. In a male fruit fly, it is the size of the genitals that is resistant to dietary restriction. This is achieved by the male fruit fly reducing expression of a key gene in their genitalia. This gene, FOXO, forms part of the insulin signaling system, which signals food levels to tissues in all animals. By lowering the production of FOXO, the genitalia are able to “ignore” hormonal signals that tell the rest of the body to grow slowly due to limited food. The ability of tissues to become insensitive to nutritional information is a characteristic of many tumors and also underlies type 2 diabetes. Our data may therefore provide insight into the origin and treatment of both conditions.

## Introduction

The ability of organisms to adjust their development., physiology or behavior in response to environmental conditions, called phenotypic plasticity, is a defining property of life. Phenotypic plasticity underlies such diverse phenomena as the relationship between childhood nutrition and adult size in humans [1], caste determination in social insects [2], and stomatal opening and closing on the leaves of plants [3]. The past 20 years have seen great progress in understanding the molecular and developmental mechanisms by which the environment influences phenotype [4]–[6]. This has been accompanied by an increasing awareness of the central role phenotypic plasticity plays in evolution [7], [8]. Nevertheless, we know almost nothing of how the *extent* of phenotypic plasticity is regulated. Why are some traits or individuals highly sensitive to an environmental variable while other traits or individuals are less sensitive?

One of the most familiar and important examples of phenotypic plasticity is the response of body and organ size to changes in developmental nutrition, here referred to as *nutritional plasticity*. In animals as diverse as humans and flies, malnutrition during development reduces adult body size [9]–[11]. This is typically accompanied with a corresponding reduction in adult organ size, ensuring that organ size scales with body size and maintaining organismal integrity [12]. Nevertheless, not all organs show the same sensitivity to changes in developmental nutrition as the body as a whole. Some traits, such as the mammalian brain, show relatively low levels of nutritional plasticity [10], and are approximately the same size in large and small individuals [13]. Other traits, particularly secondary sexual characteristics used by males to attract mates, may show relatively high levels of nutritional plasticity and are proportionally larger in large individuals compared to small individuals [14]. Differences among organs in their relative nutritional plasticity are therefore critical to regulating body proportion across a range of body sizes. Body proportion is in turn critical to the maintenance of organismal form and function.

Work over the last twenty years has identified the insulin/IGF-signaling (IIS) pathway as the major signaling pathway coordinating growth with nutritional conditions in all animals [15]–[17]. IIS activity is regulated by the nutrition-dependent release of insulin-like peptides which binds to the insulin receptor (Inr) of dividing cells. This initiates a phospho-kinase signal transduction cascade that ultimately regulates cell growth and division. This regulation is both through activation of growth promoters such as RAS/MAP kinase [18] and through the suppression of growth inhibitors such as the forkhead transcription factor FOXO [19]–[21] and TSC1/2 [22]. One appealing but untested hypothesis, therefore, is that differences among organs in their nutritional plasticity are a consequence of differences in the way they employ or regulate the IIS pathway [23]–[26].

Here we use the fruit fly, *Drosophila melanogaster*, to identify the mechanisms that regulate the degree of an organ's phenotypic plasticity with respect to developmental nutrition. In *Drosophila*, most morphological traits share the same nutritional plasticity as the body as whole. However, the male genitalia are remarkably resistant to changes in developmental nutrition – like the mammalian brain they are more or less the same size in large and small individuals [12]. This phenomenon is shared among most arthropods [27] although its evolutionary explanation remain controversial [28]. We show that the reduced nutritional sensitivity of the genitalia is a consequence of their reduced insulin-sensitivity, and demonstrate that one way insulin-sensitivity is regulated is by expression of the forkhead transcription factor *FOXO*. *FOXO* expression is necessary to maintain organ-specific differences in nutritional-plasticity and insulin-sensitivity, while organ-autonomous changes in *FOXO* expression are sufficient to autonomously alter an organ's nutritional-plasticity and insulin-sensitivity.

## Results

### Drosophila genitalia are nutrition- and insulin-insensitive

**Figure 1:**
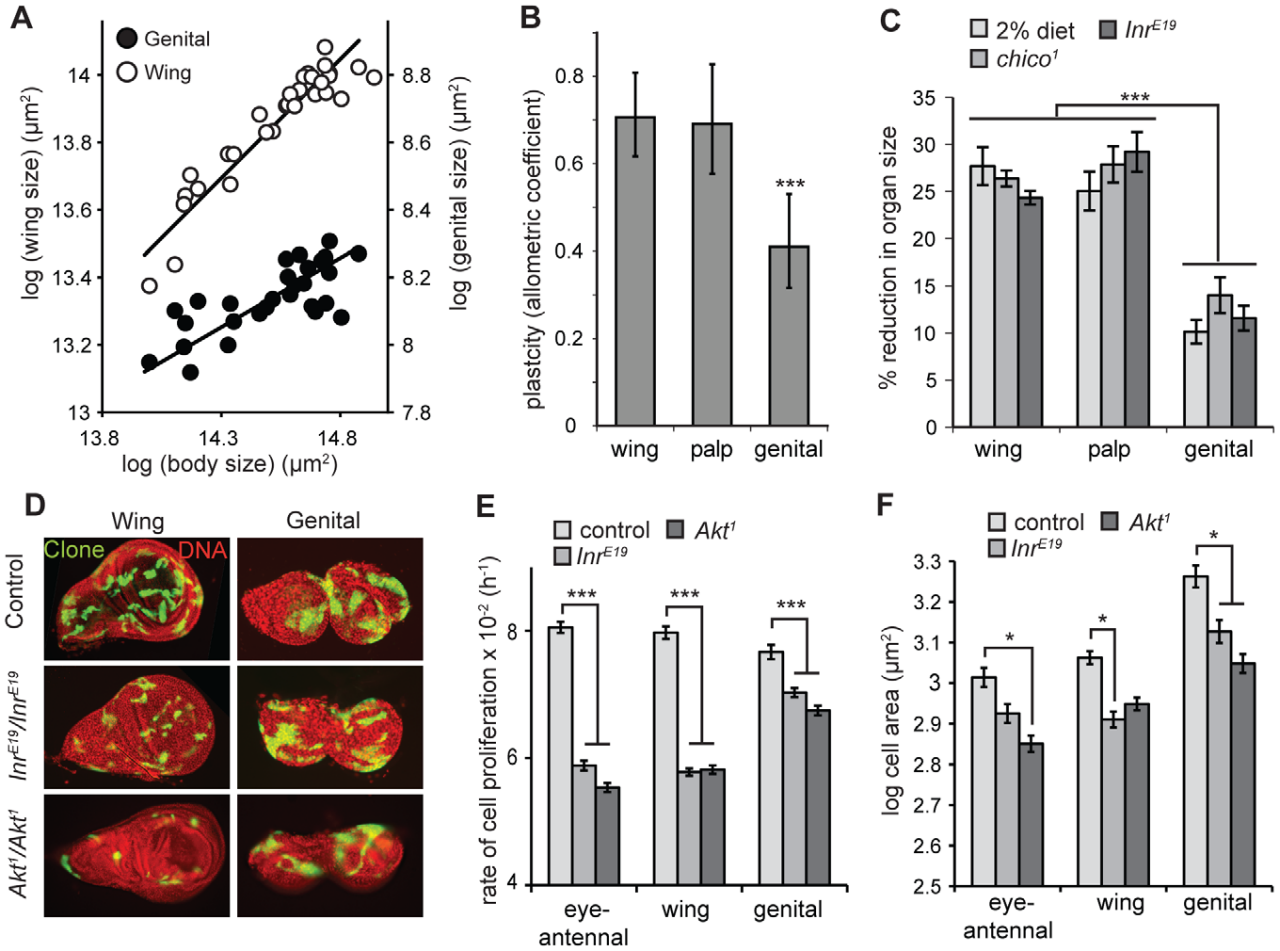
The genitalia of male *Drosophila* are nutrition- and insulin-insensitive. (A) The scaling relationship for male genital (closed circles) and wing size (open circles) against body size, where size variation is due to variation in developmental nutrition. Each line is the standardized major axis and the slope of this line – the allometric coefficient – captures the nutritional plasticity of wing and genital size relative to the nutritional plasticity of body size. (B) The allometric coefficient is significantly lower for the male genitals than for the wings or the maxillary palps, indicating a reduced nutritional plasticity. *** common slope test, *p*<0.001. (C) Flies that are homozygous for mutations of *Inr* or its substrate *chico* show a significantly smaller reduction in genital size than wing or maxillary palp size, relative to wild-type controls, genocopying starvation (2% diet) (*** Tukey HSD, *P*<0.001 for all). (D) 48 h wild-type, *Inr^E19^* and *Akt^1^* clones in wing and genital discs. Within genotypes, discs are from the same fly. Mutation of *Inr* or *Akt* has a greater effect on clone size in the wing disc than in the genital disc. Clones were induced by the MARCM system and express GFP. (E) *Inr^E19^* and *Akt^1^* mutant clones proliferate at a slower rate in the eye-antennal and wing imaginal disc than in the genital imaginal disc (*** Tukey HSD, *P*<0.001 for all). (F) Cell size within *Inr^E19^* and *Akt^1^* mutant clones is reduced by more-or-less the same degree in all discs (* Tukey HSD, *P*<0.05, non-significant comparisons not shown) Error bars are 1 standard error.

We used the *allometric coefficient* to compare the nutritional plasticity of different organs within the *Drosophila* body. The allometric coefficient (*b*) is the slope of the linear scaling relationship between two traits plotted on a log-log scale; that is where log *(trait 1 size)* = *b* log *(trait 2 size)*+*c*. The coefficient gives the extent to which variation in the size of *trait 1* is accompanied by variation in the size of *trait 2*. When size variation is due to variation in developmental nutrition, the allometric coefficient captures the nutritional plasticity of *trait 1* relative to the nutritional plasticity of *trait 2* [12]. A plot of organ size against body size for adult flies reared under a range of nutritional conditions (Figure 1A) shows that the male genitalia, as measured by the size of the genital arches, have a lower allometric coefficient, and hence lower nutritional plasticity, than other organs (Figure 1B).

The IIS pathway is the major regulator of size with respect to nutrition in all animals. The low nutritional plasticity of the genitalia in *Drosophila* may therefore be a consequence of their relative insensitivity to change in insulin signaling. This could be because the developing genitalia are exposed to elevated levels of circulating insulin-like peptides (dILPs) even when nutrition is low. dILPS are released into the hemolymph from insulin-producing cells (IPCs) in the brain, although it is possible that their distribution is modified by localized production of dILPs [29] or localized reduction of dILP-binding protein *Imp-L2* [30]. Alternatively, the genitalia may show organ-autonomous insensitivity to reduced levels of Inr activity.

Several pieces of evidence suggest that the nutritional-insensitivity of the genitalia reflects a reduction in their organ-autonomous response to changes in Inr activity. First, mutations of *Inr* (*Inr^E19^*) and its substrate *chico* (*chico^1^*) genocopy starvation and result in a more substantial reduction in the size of the wing and maxillary palp than the genitalia (Figure 1C). Second, this size effect is organ autonomous. A prior study used clonal analysis to generate maxillary palps and genitalia that were homozygous for *chico^1^* on one side of the body and heterozygous for *chico^1^* on the other. Genital arches consisting of mutant *chico^1^* clones were 16% smaller than paired genital arches on the same male [26]. In contrast, maxillary palps consisting of mutant *chico^1^* clones were 45% smaller than paired palps on the same male [26]. Third, organ-autonomous mutation of *Inr* has less of an inhibitory effect on the rate of cell proliferation in the genital discs than other discs. We used the MARCM system [31] to measure the rate of cell proliferation in *Inr* -mutant (*Inr^E19^*) and wild-type control clones generated in the imaginal discs of late first-instar larvae. While mutation of *Inr* decreased the rate of cell proliferation for clones in all the discs, the suppressive effect was significantly greater in the wing and eye-antennal discs than in the genital disc (Figure 1E). In contrast, the effects of *Inr* mutation on cell size was the same for all imaginal discs, with a reduction in cell cross-sectional area of ∼10%, (not significant for the eye-antennal imaginal disc) (Figure 1F). Collectively, these data suggest that the low nutritional plasticity of the genitalia is consequence of their relative insensitivity to the effects of insulin-signaling on cell proliferation rather than cell size [26].

### Insulin sensitivity is regulated downstream of Inr in the IIS pathway

These data suggest that the mechanism that reduces the genitals' response to changes in IIS and account for their reduced nutritional plasticity act downstream of *Inr* in the IIS pathway. The logic for this deduction is as follows. Because nutritional-insensitivity of the genitalia appears to reflect a reduction in their organ-autonomous response to changes in Inr activity, the mechanisms that regulate this insulin-insensitivity should lie within the insulin-signaling pathway itself. These mechanisms modify systemic inputs into the insulin-signaling pathway into organ-specific outputs. One method to identify where in the IIS pathway this mechanism acts is to perturb the IIS pathway at different points and assay the size effect on the genitalia compared to other organs, in well fed larvae. If the perturbation acts upstream of the mechanisms that regulate insulin-sensitivity, the perturbation will have less of an effect on the size of the genitalia compared to other organs and genocopy starvation. Conversely, if the perturbation does not act upstream of the mechanisms that regulate insulin-sensitivity the perturbation will have the same effect on the size of the genitalia as other organs. Since mutation of *Inr* and *chico* had less of an effect on the size of the genitalia than other organs, it follows that the mechanisms that account for this reduced sensitivity lie downstream of *Inr* and *chico* on the IIS pathway.

### Insulin sensitivity is regulated at FOXO

**Figure 2:**
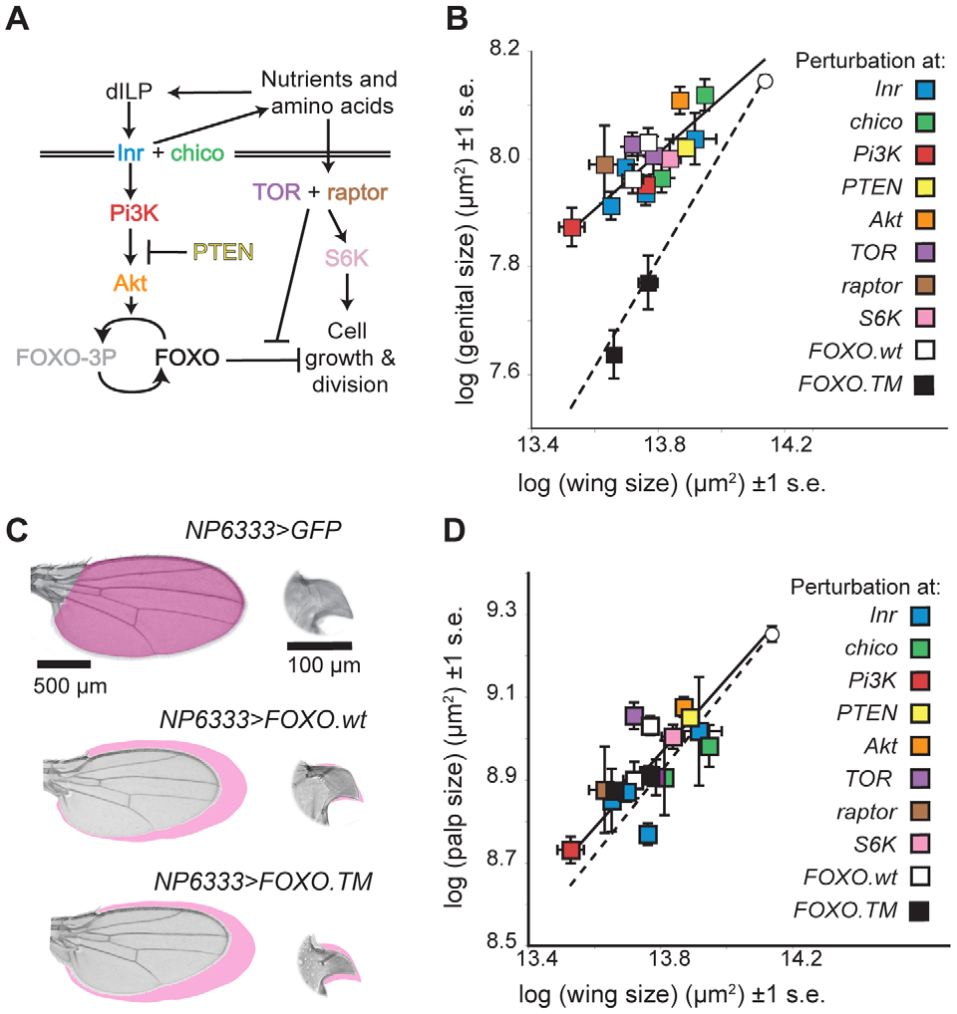
The mechanisms that reduce the insulin sensitivity of the genitalia act at FOXO in the IIS pathway. (A) The insulin-signaling pathway. (B) The effect of different IIS pathway mutations/perturbations on wing and genital size. White circle is well-fed wild-type size, and dotted line indicates where perturbation reduces genital and wing size equally. Perturbations of IIS upstream of activated FOXO, including expression of wild-type FOXO (FOXO.*wt*) (white squares) causes less of a size reduction of the genitalia than the wing of well fed flies and genocopy dietary restriction. In contrast, expression of constitutively active FOXO (*FOXO.TM*) (black squares) causes an equal reduction in both organs. Multiple markers of the same color refer to different perturbations of the same gene. (C) The effect of *FOXO.wt* and *FOXO.TM* expression on wing and genital arch size. Note that only expression of *FOXO.TM* causes a substantial reduction in genital size. Magenta shading shows area measured on control organ. Within an organ, all images are at the same scale. (D) In contrast, all these IIS pathway mutations/perturbations, including expression of *FOXO.TM* (black square) cause a more-or-less equal reduction in the size of the maxillary palps and the wings. Error bars are 1 standard error.

To determine where in the IIS pathway the mechanisms that regulate insulin-sensitivity act, we used a variety of genetic method to systematically perturb signaling at genes increasingly downstream in the IIS pathway (Figure 2A). For each perturbation we assayed whether the relative reduction in size of the genitalia compared to the wings genocopied starvation; that is whether the perturbation had less of an effect on the size of the genitalia than on the size of the wing (Figure 2B). For positive regulators of IIS we perturbed signaling either using mutation or by driving UAS-mediated expression of RNAi or dominant-negative constructs using the disc-specific GAL4-driver *P{GawB}NP6333* (here referred to as *NP6333*). For negative regulators of IIS we perturbed signaling by driving UAS-mediated expression of the gene, again using *NP6333*.

Perturbation at Chico, phosphoinositide 3-kinase (PI3K) 92E, PTEN, TOR, raptor (a co-factor of TOR), S6 Kinase (S6K) and Akt all genocopied dietary restriction and had less of an effect on the size of the genitalia than on the wings (Figure 2B). In contrast, all these perturbations had the same effect on the size of the maxillary palps as the wings (Figure 2C). This suggests that the mechanisms that reduce insulin-sensitivity in the genitalia lie downstream of these genes in IIS pathway.

The next gene downstream of Akt in the canonical IIS pathway is the Forkhead Box O transcription factor (FOXO) (Figure 2A). FOXO is a negative growth regulator, albeit one that is only activated when IIS is low [32]. When IIS is high, FOXO is phosphorylated by Akt. This disrupts DNA binding and causes FOXO to translocate to the cytoplasm [19]–[21], [33]. A decline in IIS leads to de-phosphorylation of FOXO, which accumulates in the nucleus and initiates the transcription of growth inhibitors, for example 4EBP [21]. Increased expression of FOXO decreases body and organ size [21]. Loss of FOXO, however, has no obvious effect on size in well-fed flies [19], presumably because in such flies FOXO would otherwise be deactivated by high IIS. In contrast, when IIS is low, for example in *Inr, chico* and *Akt* mutants, loss of FOXO attenuates any decrease in size [19]. FOXO is therefore necessary and partially sufficient for growth suppression in IIS mutant and starved flies [19].

Over-expressing *FOXO* in the imaginal discs using *NP6333* genocopied dietary restriction, reducing the size of the adult wings and maxillary palps by ∼30% but only reducing the size of the genitalia by ∼15% (Figure 2B). This was not because the GAL4 driver expressed weakly in the genital disc: *NP6333* drives expression of GFP in the wing and the genital discs equally (Figure S1). In contrast, using *NP6333* to drive expression of constitutively activated forms of FOXO (*FOXO.TM*) in the imaginal discs of well-fed larvae had the same effect on the genitalia, wing and maxillary palps, causing a ∼30% reduction in size (Figure 2B and 2C). *FOXO.TM* is mutated at the three Akt-phosphorylation sites T44, S190 and S259. This permits insulin-insensitive nuclear transport and so its activity can not be suppressed by Akt [34].

The genitalia are therefore less sensitive to increased expression of *FOXO.wt* but not *FOXO.TM* when both are expressed using the same driver, while the wings and maxillary palps are equally sensitive to both. This suggests that the genitalia are better able to maintain phosphorylation at FOXO's AKT-phosphorylation sites, and hence limit FOXO's transcriptional activity, even when nutrition and IIS is low.

**Figure 3:**
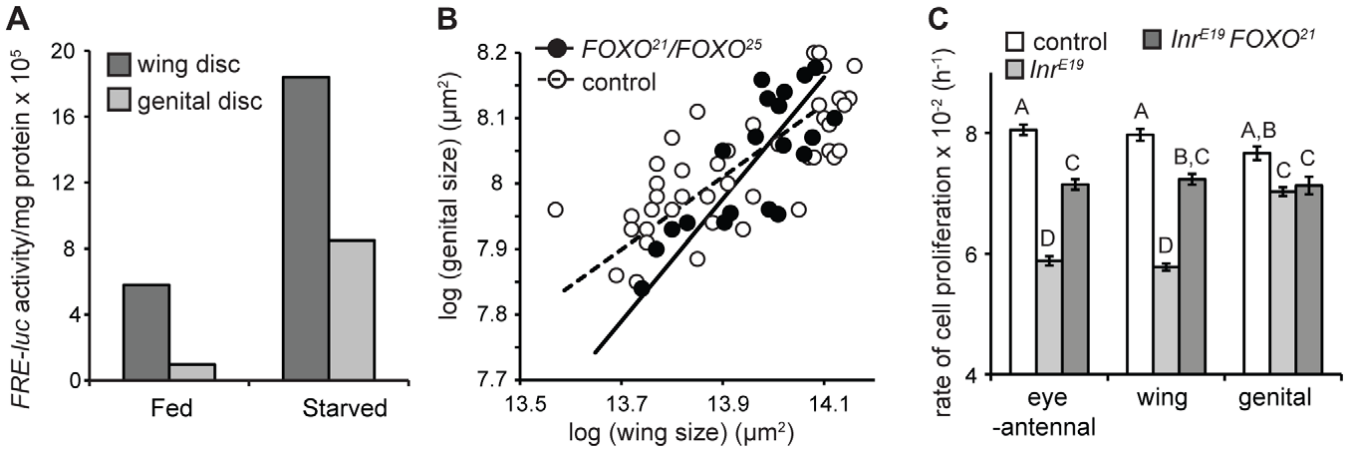
FOXO is necessary to maintain organ-specific nutritional plasticity and insulin sensitivity. (A) The genital imaginal discs of fed larvae have low levels of activated FOXO compared to the wing discs and show less of an increase in activated FOXO after 24 hours of starvation. (B) The scaling relationships between wing and genital size in wild-type and *FOXO*-mutant flies, where variation in size is due to variation in developmental nutrition. In control flies, starvation has less of an effect on genital size than wing size (slope = 0.55, 95% C.I. = 0.45–0.68). In contrast, starvation has does not have a significantly different effect on the size of the wing and genital in *FOXO*-mutant flies (slope = 0.93, 95% C.I. = 0.69–1.27). (B) Mutation of *FOXO*-attenuates the effect of *Inr*-mutation on the rate of cell proliferation in clones generated in the eye-antennal and wing imaginal discs, but not in the genital discs. The rate of cell proliferation in *Inr-FOXO* double mutant clones is not significantly different among discs (mixed model ANOVA, *P* = 0.771). Columns with the same letter are not significantly different (Tukey HSD, *P*>0.05). Error bars are 1 standard error.

To confirm this, we measured FOXO activity in the wing and genital imaginal discs of fed and starved third instar larvae using the *FRE-luciferase* (*FRE-luc*) reporter construct [35]. The construct comprises the firefly *luciferase* gene under the transcriptional control of the herpes simplex minimal promoter and 8 direct repeats of the FOXO Response Element (FRE) [35]. FOXO activity can therefore be assayed by measuring luciferase activity. In both starved and fed larvae FOXO activity was higher in the wing than in the genital discs and the increase in FOXO activity upon starvation was greater in the former than in the latter (Figure 3A). Thus the genital discs are better able to limit FOXO activity when nutrition, and presumably IIS, is low.

### FOXO is necessary to maintain organ-specific difference in nutritional- and insulin-sensitivity

If variation among organs in their nutritional- and insulin-sensitivity is mediated by FOXO, then loss of FOXO should result in all organs showing the same level of nutritional- and insulin-sensitivity. To test this we examined the nutritional plasticity of the wings, palps and genital in flies mutant for FOXO (*FOXO^21^/FOXO^25^*) [19]. *FOXO^21^/FOXO^25^* mutants produce no detectable protein [36] and are assumed to be nulls [19]. Nevertheless, there does appear to be some residual binding of FOXO to DNA in these flies [36], so we will refer to these flies as *FOXO*-mutant rather than *FOXO*-null [37].

In wild-type flies reared under a range of nutritional conditions, a log-log plot of genital size against wing size has a gradient less than 1, indicating that for any reduction in wing size there is less of a reduction in genital size (Figure 3B). However, for *FOXO* mutants, this plot has a gradient not significantly different from 1, indicating that the effect of nutrition on organ size is the same in the wings and the genitalia (Figure 3B). Thus FOXO appears necessary to maintain differences in nutritional plasticity between the wing and the genitalia.

We used clonal analysis to determine whether FOXO is necessary to maintain the organ-specific response of cell proliferation to changes in IIS. Previous studies have demonstrated that loss of *FOXO* suppresses growth-deficient phenotypes of *Inr* mutants [19], [36]. Consistent with these studies we found that the rate of cell division in *Inr*-mutant clones in the imaginal discs was partially rescued if these clones were also mutant for *FOXO* (*FOXO^25^*) (Figure 3C). However, this rescue was only seen in the wing and eye-antennal discs. *Inr-FOXO* double mutant clones in the genitalia proliferated at the same rate as *Inr* mutant clones (Figure 3C). The result was that the rate of cell proliferation was the same in *Inr-FOXO* double mutant clones in the wing, eye-antennal and genital imaginal discs. In other words, mutation of FOXO reduces the insulin-sensitivity of cell proliferation in the eye-antennal and wing discs so that it is equal to the insulin-sensitivity of cell proliferation in the genital disc. Thus FOXO appears necessary to maintain the organ-specific response of cell proliferation to changes in IIS.

### Limited FOXO activity in the genitalia is not due to increased Akt activity

Collectively these data suggest that the reason the male genitalia of *Drosophila* have a limited response to changes in nutrition and IIS is because they are able to limit the transcriptional activity of FOXO when nutrition and IIS is low, effectively restricting the genitals' size-response to one that is independent of FOXO. Other organs only show this reduced sensitivity to changes in nutrition and IIS when mutant for *FOXO*.

One mechanisms by which the genitalia could limit the transcriptional activity of FOXO when IIS signaling is low is if the suppressor of FOXO, Akt, were unusually active in the genital imaginal disc. If this were the case, then complete loss of Akt should remove this differential activity and reduce growth equally in the genital, wing and eye-antennal discs. To test this we generated clones of *Akt* null cells (*Akt^1^*) in the developing imaginal discs [38]. Loss of *Akt* had less of an effect on the rate of cell proliferation in the genital discs compared to the wing and the eye-antennal discs (Figure 1E). In fact, the effect on cell proliferation in the genital disc compared to the wing and eye-antennal discs was the same as for mutation of *Inr*. In contrast, loss of *Akt* had the same effect on cells size in all discs (Figure 1F). Thus the mechanisms that reduce the insulin-sensitivity of the genitalia are not contingent on heightened activity (or even presence) of Akt in the genitalia. Further, mutation of *FOXO* attenuated the effects of *Akt* mutation on cell proliferation in the eye-antennal and wing discs but not in the genital disc, with the rate of cell proliferation in *Akt-FOXO* double mutant clones more-or-less the same in all three disc types (Figure S2). Thus the organ-specific effects of *Akt* mutation, as for the organ-specific effects of *Inr* mutation, are FOXO dependent.

### Limited FOXO activity in the genitalia is correlated with reduced FOXO expression

A second mechanism by which the genitalia could reduce levels of activated FOXO when IIS is low is through reduced expression of *FOXO* itself. Organs with low expression levels of *FOXO* might have less FOXO available to inhibit growth, and would require less activated Akt to phosphorylate what little FOXO there is. This would account for differences among organs in their response to increased expression of *FOXO*: organs with low levels of endogenous FOXO may be more able to deactivate any additional FOXO, there-by reducing the effect on size.

**Figure 4:**
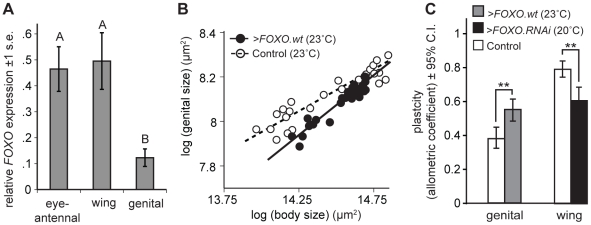
Organ-specific nutritional plasticity is regulated by differential expression of *FOXO*. (A) The genital imaginal discs express an unusually low level of *FOXO* relative to the wing and eye-antennal imaginal discs (* Tukey HSD, *P*<0.05). (B) The scaling relationship between genital and body size for flies reared under different nutritional conditions. Driving expression of *FOXO.wt* in the genitalia (*NP6333>FOXO.wt*) increases the slope of the scaling relationship, and hence the genitalia's nutritional plasticity, relative to wild-type controls (*NP6333>GFP*). (C) Up-regulating *FOXO* expression in the genitalia (*NP6333>FOXO.wt*) significantly increases their nutritional plasticity while down-regulating *FOXO* expression in the wing (*NP6333>FOXO.RNAi*) significantly decreases their nutritional plasticity, compared to wild-type controls (*NP6333>GFP*) (** Common Slope Test, *p*<0.01). Because we used the temperature-dependence of GAL4 activity to modulate expression, experimental temperatures are indicated in parentheses (see Materials and Methods). Controls were reared at the experimental temperature.

To examine this we used quantitative RT-PCR (qPCR) to measure the expression of *FOXO* in the developing genital, wing and eye-antennal imaginal discs. We found that the genital discs express significantly lower levels of *FOXO* compared to other organs (Figure 4A).

### Up- or down-regulating FOXO expression is sufficient to alter organ plasticity

If reduced *FOXO* expression were indeed the mechanism by which the genitalia reduce their insulin-sensitivity and nutritional plasticity, then increasing expression of *FOXO* in the genitalia should increase their size response to changes in nutrition. Conversely, decreasing *FOXO* expression in the wings should reduce their size response to changes in nutrition.

To test this we altered expression of *FOXO* in the developing wing and genital imaginal discs and assayed the extent to which adult wing and genital size responded to changes in developmental nutrition, that is their nutritional plasticity. We used *NP6333* to drive *FOXO.wt* and *FOXO.RNAi* expression, increasing and decreasing *FOXO* expression respectively (Figure S3). We measured nutritional plasticity as the slope of the scaling relationship between organ size and body size (the organ's allometric coefficient) where variation in size is a consequence a variation in developmental nutrition.

Consistent with our hypothesis, increasing the expression of *FOXO* in the genitalia increased their nutritional plasticity compared to controls (Figure 4B and 4C). Conversely, decreasing the expression of *FOXO* in the wings decreased their nutritional plasticity (Figure 4C). Expression of *FOXO.RNAi* in the genitalia reduced *FOXO* expression to immeasurable levels but did not, however, further reduce their nutritional plasticity (*p* = 0.622). The effects of *FOXO* expression on plasticity were organ autonomous. *NP6333* does not drive expression in the leg imaginal discs and the nutritional plasticity of the legs were unaffected by changes in *FOXO* expression in other imaginal discs (Figure S4). Changing in *FOXO* expression in the imaginal discs also did not influence final body size (Figure S4).

### There is a non-linear relationship between FOXO expression and organ plasticity

**Figure 5:**
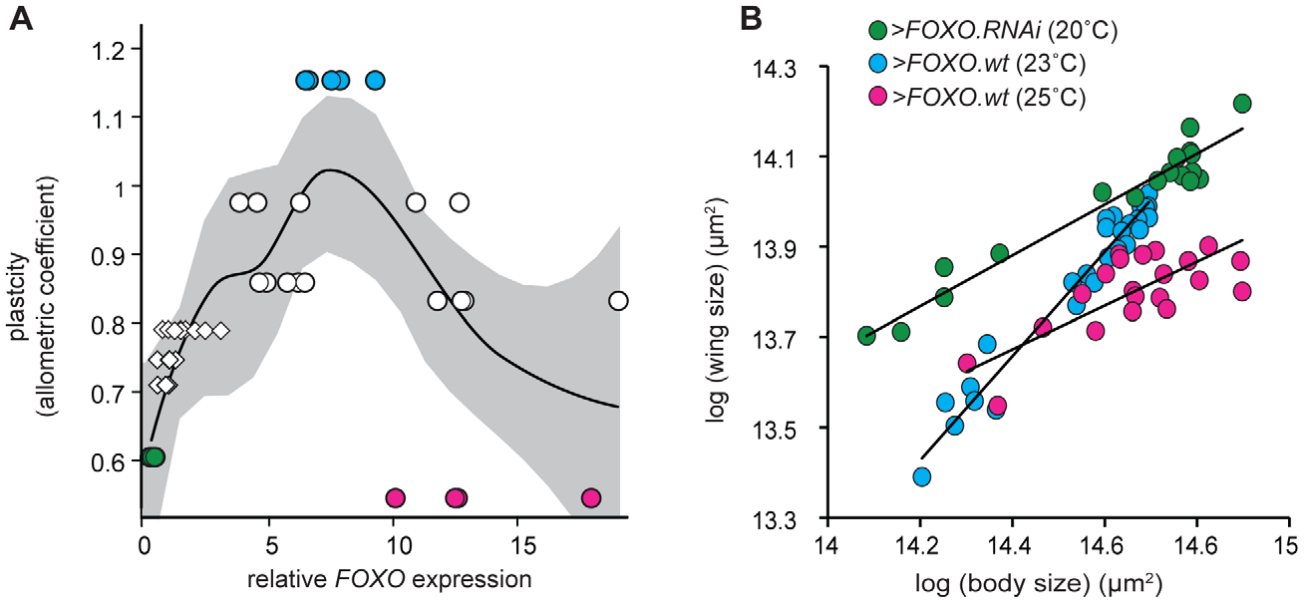
There is a non-linear relationship between *FOXO* expression and nutritional plasticity. (A) A moderate increase in *FOXO* expression in the wing results in an increase in its nutritional plasticity, while a more substantial increase causes a decrease in its nutritional plasticity. Expression levels are normalized to wild-type expression at 25°C. Plasticity is the allometric coefficient of the wing-pupal scaling relationship, uncorrected for temperature. Line is quadratic regression for raw data, gray shading is 95% CI, N = 49. Circles indicate flies in which we have manipulated *FOXO* expression using UAS-GAL4, diamonds indicate wild-type flies reared at different temperatures (see Figure S5). (B) The nutritional static allometry of wings with different levels of *FOXO* expression shows that at very high levels of *FOXO* expression (>*FOXO.wt* at 25°C), wing size is small even in well-fed flies with large bodies, while at very low levels of *FOXO* expression (*>FOXO.RNAi* at 20°C) wing size is large even in poorly-fed flies with small bodies. Note that in both situations the nutritional plasticity of the wing is reduced. Data is normalized to control for the effects of temperature on scaling (see Materials and Methods). Marker color in (B) refer to data points in (A).

To further explore how FOXO influences insulin-sensitivity and nutritional plasticity, we manipulated expression of *FOXO* in the wing by exploiting the temperature dependence of GAL4 activity [39]. We reared *NP6333>FOXO.wt* larvae at increasingly higher temperatures (17–25°C), which resulted in increasingly elevated levels of *FOXO* expression in their wing discs (Figure S5). Surprisingly, while a moderate increase in *FOXO* expression increased the nutritional plasticity of the wing (*>FOXO.wt* at 23°C), substantial increases in *FOXO* expression (*>FOXO.wt* at 25°C) reduced plasticity to a level below that observed when *FOXO* expression is down-regulated (*>FOXO.RNAi* at 20°C) (Figure 5A). These effects were not due to the effects of temperature on nutritional plasticity: nutritional plasticity of wild-type control wings slightly decreased with an increase in temperature, and this was accompanied by a corresponding decrease in the expression of *FOXO* (Figure S6). Further analysis revealed that very high and very low levels of *FOXO* expression affected nutritional plasticity in different ways (Figure 5B). A reduction in *FOXO* expression reduced wing plasticity by inhibiting a decrease in wing size in poorly-fed flies, with flies maintaining a large wing size across a range of nutritional conditions. In contrast, a substantial increase in *FOXO* expression reduced wing plasticity by inhibiting an increase in wing size in well-fed flies, with flies having reduced wings across a range of nutritional conditions.

## Discussion

### *FOXO* and plasticity

These data support the hypothesis that the extent of nutritional plasticity of organ size in *Drosophila* is regulated by FOXO. The genitalia of *Drosophila* show low levels of nutritional plasticity and are able to maintain their size even in larvae that are food-restricted. The mechanisms that account for this reduced plasticity are dependent on and act at FOXO in the IIS pathway. FOXO is a growth inhibitor that is deactivated by IIS when developmental nutrition is high but becomes active as the level of nutrition and IIS activity falls. The growing genitalia appear to attenuate their size-response to changes in nutrition and IIS by expressing only low levels of *FOXO*, thereby limiting the activation of FOXO in conditions of low nutrition.

Implicit to this model of plasticity regulation is that the IIS and FOXO affect organ size by suppressing growth when nutrition is low and permitting growth when nutrition is high. It follows that there are mechanisms other than IIS that promote growth in the imaginal discs, the downstream effects of which are suppressed by FOXO in low nutritional conditions. Indeed, the fact that cells lacking *Inr* or *Akt* are able to proliferate relatively efficiently in the genital discs, and in wing and eye-antennal discs with mutant *FOXO*, indicate that growth can occur independently of IIS. It is possible, therefore, that the low nutritional plasticity of the genitalia reflects the genital-specific activation, rather than de-repression, of other growth-promoting pathways when IIS is low. Our data suggest that this is not the case. We found that FOXO expression is necessary to maintain the differential response of discs to changes in nutrition and IIS, and that decreasing FOXO expression is sufficient to reduce a disc's nutritional- and insulin-sensitivity. Thus any putative up-regulation of growth-promoting pathways in the genital discs of malnourished larvae is FOXO dependent. It is difficult to conceive of a mechanism by which lowering FOXO expression in an individual organ could promote that organ's growth in malnourished larvae, except if FOXO were acting as a nutrition-dependent growth inhibitor.

The mechanism by which FOXO regulates size explains why both low and high levels of *FOXO* expression reduce an organ's nutritional plasticity. At low levels of *FOXO* expression growth is not inhibited when nutrition and IIS is low and organs maintain a large size even in larvae that are nutritionally stressed. On the other hand, at high levels of *FOXO* expression there may be insufficient activated Akt to phosphorylate and deactivate FOXO even when IIS is high, and organs maintain a small size even in larvae that are well-fed. This reduction in organ size is due to the suppressive effects of activated FOXO on cell proliferation, but may also be a consequence of activated FOXO increasing apoptosis [40]. Thus nutrition appears to modulate organ size within a specific range, with *FOXO* expression regulating how much of this range is realized across nutritional conditions. What defines the limits of this range is unclear. Cells lacking *Inr* and *Akt* continue to proliferate, albeit at a reduced rate, confirming the existence of growth-promoting mechanisms that are IIS independent. ‘Minimum’ organ size may therefore reflect the residual activity of these growth-promoting mechanisms when FOXO is maximally activated. Conversely, ‘maximum’ organ size may reflect the activity of these growth-promoting mechanisms when FOXO is absent.

*FOXO* expression is both sufficient and necessary to generate organ-specific differences in nutrition- and insulin-sensitivity. However, increasing *FOXO* expression in the genital discs did not elevate their nutritional plasticity to that of the wing. This may be a consequence of the non-linear relationship between *FOXO* expression and plasticity – a more moderate increase in *FOXO* expression in the genital discs may elevate their nutritional plasticity further. Nevertheless, additional processes might limit the nutritional plasticity of the genitalia, independent of *FOXO* expression. For example, it is possible that factors apart from Akt suppress the activity of FOXO in the genital discs of malnourished larvae. These factors would presumably act by phosphorylating FOXO at the same sites as Akt, since the genitalia do not appear to be resistant to activated FOXO that is mutant at these sites (FOXO.TM). Such factors exist in mammals (serum/glucocrticoid-induced kinase, SGK [33]), but have not yet been identified in *Drosophila*. Further, nutritional insensitivity in mammals appears to be conferred by localized production of insulin-like growth factors, specifically in the CNS [41]. Our data suggest that the nutritional-insensitivity of the genitalia can be wholly explained by their insensitivity to changes in Inr activity (Figure 1C). Even so, it is possible that local sources of dILPs may also ameliorate the effects of reduced nutrition on the systemic supply of dILPs from the IPCs to individual organs [29], [42]. Examining the insulin-sensitivity of discs cultured *in vitro* would test this hypothesis directly.

It will also be interesting to explore the role of TOR-signaling in regulating disc-specific nutritional sensitivity. We found that the genitalia were relatively insensitive to changes in raptor, TOR and S6K activity (Figure 2B). The loss of disc-specific nutritional sensitivity in flies mutant for *FOXO* suggest that FOXO also plays a role in regulating a disc's response to changes in nutrition via TOR-signaling. However, whilst there is considerable crosstalk between the IIS and TOR signaling pathways [43]–[45], it is not immediately clear how this regulation would be achieved.

A recent study by Cheng et al revealed that anaplastic lymphoma kinase (Alk) plays a key role in limiting the response of the CNS to changes in developmental nutrition in *Drosophila* [46]. Larvae that are nutritionally restricted late in larval development are able to continue growth of CNS in conditions that inhibit growth of the body as a whole. Alk is a receptor tryrosine kinase that activates PI3K independently of Inr, allowing PI3K-regulated growth in the CNS even when nutrition and Inr activity is low [46]. Like the CNS, the imaginal discs are also able to grow when nutrition and Inr activity is restricted late in larvae development, albeit at a reduced rate [23], [47], and this may also be a consequence of Alk activity. However, Alk does not appear to account for variation among discs in their insulin-sensitivity. This is because Alk acts upstream of PI3K to regulate insulin-independent growth: the CNS is insensitive to a reduction in Inr activity but not to a reduction in PI3K or Akt activity [46]. In contrast, our data indicate that final genital size is relatively insensitive to a reduction in both PI3K and Akt activity, suggesting that the mechanisms that regulate this insensitivity lie downstream of these genes in the IIS. Thus there appears to be at least two mechanisms that limit nutritional sensitivity in *Drosophila* organs: Alk-signaling, as observed in the CNS, and low levels of *FOXO* expression, as observed in the genital discs.

Work over the last decade has established FOXO as a major regulator of longevity, diabetes, and organ and body size. Our study expands this role to include regulation of nutritional plasticity and insulin-sensitivity. However, FOXO may be a more general plasticity gene [48]. The male genitalia of *Drosophila* show reduced plasticity not only in response to developmental nutrition but also developmental temperature and density [12]. *FOXO* lies at the nexus of a number of other signaling pathways involved in size regulation [49], including the Wingless [50], JNK [40], HIF [51] and Hippo/MST signaling pathways [52]. It is possible, therefore, that changes in *FOXO* expression is a common mechanism by which organs regulate their response to environmental factors that reduce size. Further, if genetic variation in size is a consequence of allelic variation in these different signaling pathways, then low levels of *FOXO* may also limit an organ's response to genetic factors that reduce size.

### FOXO and morphological scaling

By altering an organ's nutritional plasticity we affected how that organ's size scaled with body size, as both varied with nutritional condition. The scaling relationship between organ and body size controls body proportion and defines the shape of an animal [53]. Evolutionary diversity is dominated by variation in shape and changes in morphological scaling is one of the primary mechanisms by which this variation is generated [54], [55]. Indeed, the phenomenon of scaling and its developmental regulation has intrigued some of the greatest minds in evolutionary biology over the last 100 years [54]–[57]. Knowledge concerning the proximate mechanisms that produce morphological scaling relationships is therefore central to understanding of the development and evolution of morphology. Our study identifies *FOXO* as a key regulator of morphological scaling in *Drosophila*. However, the importance of nutrition as a regulator of size in animals and the evolutionary conservation of the IIS suggests that *FOXO* may be a proximate target of selection on morphological scaling in animals in general.

The non-linear relationship between *FOXO* expression and nutritional plasticity means that ostensibly similar scaling relationships may be achieved either through increases or decreases in *FOXO* expression. In *Drosophila*, nutritional-insensitivity of the genitalia is achieved through a reduction in *FOXO* expression, with flies maintaining a near maximum genital size even in poorly-fed individuals. In the horned beetle, *Onthophagus nigriventris*, horn size in small males and females is also nutritionally-insensitive and is more-or-less constant across a range of body sizes [58]. However, in this case it is because these beetles suppress horn growth and maintain a minimum horn size even in better-fed individuals. Such a phenotype would result if *FOXO* expression were relatively high in the developing horns of small males and females. Indeed, this is supported by the finding that expression levels of *Inr*, a transcriptional target of *FOXO*, are elevated in these horns [58]. Thus, while *FOXO* expression may prove to be a proximate target of selection on morphological scaling, its response to selection will depend on the nature of the selective pressure.

### The regulation and evolution of phenotypic plasticity

Plasticity is a fundamental biological process that ensures that individuals' morphology, behavior and physiology match their environment. An essential aspect of this process is how these pathways are modified to either amplify or attenuate the environmental signal to which an individual is responding, thereby modulating the extent of the plastic response. Understanding the mechanisms that regulate the extent of trait plasticity is important for two reasons:

First, an understanding of how phenotypic plasticity is regulated has important consequences for the study of diseases that result from changes in plasticity. One particularly relevant example is type 2 diabetes, characterized by a reduction in insulin-sensitivity. Interestingly, *Foxo1* expression appears to be a positive regulator of insulin-sensitivity in mammalian kidney cells [59] but a negative regulator in the liver, adipocytes and pancreatic β-cells [60]. Such apparently contradictory findings provide additional support for a non-linear relationship between *FOXO* expression and nutritional- and insulin-sensitivity.

Second, phenotypic plasticity – and its inverse environmental canalization – are increasingly recognized as playing a central role in evolution. Numerous studies have demonstrated that trait plasticity varies within and between species (e.g. [61]–[64], see [48] for review) and can be altered through selection [65]–[68]. Further, plasticity may facilitate the evolution of novel traits through genetic assimilation [7], [69]. Nevertheless, the developmental mechanisms that are the target for selection on plasticity remain poorly elucidated. Without such elucidation our understanding of how these mechanisms facilitate and bias evolutionary processes will remain incomplete.

Our study provides a foundation for future research into the regulation of phenotypic plasticity. The data suggest that variation in plasticity, either between different traits within an individual, or between the same trait in different individuals and species, may be consequence of differences in the expression of genes involved in signaling environmental information to developmental (or physiological or behavioral) processes. The generality of this mechanism in regulating the extent of phenotypic plasticity, however, requires further investigation.

## Materials and Methods

### Flies

The following flies were used in this study (stock numbers are in parentheses): The GAL4-driver *P{GawB}NP6333* (113920) is expressed in the wing, eye-antennal, and genital imaginal discs and was acquired from the DGRC, Kyoto, Japan. *UAS-Akt.RNAi* (2902), *UAS-PI3K.RNAi* (38986 & 38986), *UAS-Inr.RNAi* (992 & 993), *UAS-FOXO.RNAi* (30556), and *UAS-raptor.RNAi* (13112) were from the VDRC (Vienna, Austria). *Inr^GC25^* (9554), *Inr^E19^* (9646), *UAS-GFP* (5430) UAS-*Inr.DN* (8253), *UAS-TOR.TED* (7013), *UAS-TOR.WT* (7012) and FRT82B *arm*-lacZ (7369) were from the Bloomington stock center (Bloomington, IN). *S6K^l-1^* was the kind gift of George Thomas. *chico^1^*, *FOXO^21^* and *FOXO^25^* was the kind gift of Ernst Hafen. *Akt^1^* was the kind gift of Hugo Stocker. *UAS-PTEN* was a kind gift of Bruce Edgar. *UAS-FOXO.wt* was the kind gift of Jamie Kramer. *UAS-FOXO.wt (m3-1)*, *UAS-FOXO.TM (f3-9)* and *UAS-FOXO.TM (m6-15)* were the kind gift of Marc Tater. *y,w,UAS-GFP; tub-GAL4, FRT82B, tub-GAL80* was the kind gift of Melissa Gilbert. *P{GAL4}NP6333*, UAS-FOXO.wt (Kramer), and *UAS-GFP*, were used to assay the affect of *FOXO* expression on morphological scaling, and were made coisogenic through backcrossing into a wild-type SAM background for 5 generations. *FRE-Luc* was the kind gift of Brian Staveley.

### Scaling relationships

All scaling relationships were for isogenic flies where variation in size was due to variation in developmental nutrition [70]. Flies were crossed and females allowed to oviposit in vials containing standard cornmeal/molasses medium for a 24 hour period (∼50 eggs per vial). Each vial was then left for a further 4 days, at which point the larvae in a vial were between 4 and 5 days old and showed a range of sizes. All the larvae in the vial were transferred to individual 1.5 ml microcap tubes without food and left to complete development. Because the larvae were starved at different sizes they generated adults of a similar range of sizes, where size variation was due to differences in the amount of developmental nutrition each larva received. Adults were dissected as described in [12]. Previous studies have shown thorax length to be a less than ideal proxy for overall body size [12], but that there is a tight correlation between pupal size and adult body size [71]. Consequently, we used pupal case size as a measure for body size. Digital images of pupal cases were collected and the area of the pupal case when viewed from the dorsal aspect was measured. The size of other parts of adult morphology were measured as described in [12]. Scaling relationships were fitted using the standardized major axis, and slopes were compared using the *smatr* [72] package in R [73]. Unless otherwise stated, all larvae were reared at 25°C in constant light. However, *NP6333>FOXO.RNAi* larvae were reared at 20°C, since larvae reared at higher temperatures did not eclose as adults, while *NP6333>FOXO.wt* larvae were reared at 17, 20, 23, 24 and 25°C, as a means to control the expression of *FOXO*.

For Figure 5B, the wing-body scaling relationships for *NP6333*>*FOXO.RNAi* (20°C), *>FOXO.wt* (23°C) and *>FOXO.wt* (25°C) flies were normalized for temperature using the wing-body scaling relationships of the control flies (*NP6333>GFP*) at 20, 23 and 25°C. We first transformed the data for experimental and control flies reared at 20 and 25°C so that the bivariate mean of wing and body size for the un-starved control flies was equal to that of the un-starved control flies reared at 23°C. We then used this bivariate mean as an anchor point around which we rotated the data for the 20 and 25°C experimental and control flies such that the slope of the scaling relationship for the controls flies was equal to that of the control flies reared at 23°C. In sum, these transformations resulted in a common control scaling relationship at all three temperatures, against which the experimental scaling relationships were plotted.

### Perturbation of the IIS pathway

The IIS pathway was perturbed at Inr using mutation (*Inr* ^E19^), RNAi (*NP6333>UAS-Inr.RNAi*) and by expressing a dominant negative of Inr (*NP6333>UAS-Inr.DN*); at Chico using mutation (*chico^1^*) and RNAi (*NP6333>UAS-chico.RNAi*); at PI3K using RNAi (*NP6333>UAS-Pi3K.RNAi*); at PTEN by over-expressing *pten* (*NP6333>UAS-PTEN*); at AKT using RNAi (*NP6333>UAS-Akt.RNAi*); at raptor using RNAi (*NP6333>UAS-raptor.RNAi*); at TOR by over-expressing *Tor* (*NP6333>UAS-TOR.WT* and *NP6333>UAS-TOR.TED*); at S6 Kinase by mutation (*S6K^l-1^*) and RNAi (*NP6333>UAS-S6K.RNAi*), and at FOXO by over-expressing wild-type and constitutively active FOXO (*NP6333>UAS-FOXO.wt* and *NP6333>UAS-FOXO.TM* respectively). All larvae were reared at low density on standard cornmeal/molasses medium at 25°C. Body parts were measured as described in [12].

### Clonal analysis

Clones were induced using the MARCM system and marked using GFP [31]. Flies were of the genotype *hsflp*; *tub-GAL4*, *FRT82B*, *tub-GAL80/FRT82B*, *X*, where *X* was either *arm*-lacZ (control), *Inr^E19^*, *Akt^1^*, *Inr^E19^+FOXO^21^*, or *Akt^1^+FOXO^21^*. Females were left for 2 h to clear retained eggs and then allowed to lay a 6 h cohort of larvae. Larvae were heat-shocked at 37°C for 1.5 h, 42 h after egg laying to generate mitotic clones. Clones were left to develop for ∼48 h before the wing, eye-antennal and genital imaginal discs from each larva were dissected and fixed. The timing of each dissection was recorded to calculate the precise age of clones within each larvae. Discs were dissected from eight to 10 larva for each genotype. The discs were imaged using standard methods and the number of cells within each clone was recorded. The number of clones per disc ranged from five to 30. The rate of cell proliferation for each clone was calculated as *log(N)/t* where *N* is the number of cells in each clones and *t* is the age of the clone. A mixed model analysis of variance (ANOVA) with *disc type* and *genotype* as fixed effect and *larvae* as random effect was used to estimate the mean rate of cell division for each genotype/disc type combination whilst controlling for variation in the rate of cell division among larvae. A subsequent Tukey HSD test was used to compare specific rates of cell division between specific genotype/disc-type combinations. We also estimated the size of cells within each clone by measuring their cross-sectional area at the surface of the disc. The data were again analyzed using a mixed model ANOVA to calculate mean cell size for each genotype/disc type combination. All analyses were conducted with JMP (SAS Institute).

### Quantitative real-time PCR (qPCR)

qPCR was conducted on imaginal discs from male SAM wild-type third instar larvae reared at low density on standard cornmeal/molasses medium at 25°C and dissected 39 hours after ecdysis from the second to the third larval instar. Gene expression was assayed on four to five biological replicates, using a standard curve and normalized against expression of *28S* rRNA. Primers for assaying *FOXO* expression levels were AGGCGCAGCCGATAGACGAATTTA (forward) and TGCTGTTGACCAGGTTCGTGTTGA (reverse). Primers for assaying 28S expression levels were TAACGAACGAGACTCAAATATAT (forward) and GAATGAAGGCTACATCCGC (reverse). Standard curves were generated using seven serial dilutions of total RNA extracted from 2× 1^st^ instar larvae, 2× 2^nd^ instar larvae, 2× 3^rd^ instar larvae (male), 2× pupae (male) and 2× adult flies (male). The same methods was used to assay gene expression in the imaginal discs of *NP6333>FOXO.wt*, *NP6333>FOXO.RNAi*, and *NP6333>GFP* larvae. However, because these larvae were reared at different temperatures, wing imaginal discs were dissected at a specific developmental stage (wandering) rather than a specific larval age.

### FOXO activity assay

*FRE-luc* larvae were reared on standard cornmeal medium at 25°C and staged into 4 hour cohorts at ecdysis to the third larval instar. Larvae were then reared at 25°C for an additional 15 hours before being either starved for 24 hours or left to continue feeding. Larvae were then dissected in PBS and their wing and genital imaginal discs were stored in minimal PBS at −80°C. One hundred wing and 100 genital imaginal discs from both fed and starved larvae were homogenized in 50 µl of PBS with protease inhibitor (Roche) and then centrifuged at 13,0000 rpm for 5 minutes. We then tested 10 µl of the supernatant for lucifersase activity using the Promega Luciferase Assay System. We measured the protein concentration for each sample using a standard BCA assay and normalized the luciferase activity as activity per mg.

## Supporting Information

Figure S1 *NP6333* drives expression equally in the wing and genital imaginal discs. Expression of *GFP* (assayed by qPCR) 39 hours after ecdysis from the second to the third larval instar is not significantly different in wing and genital discs of *NP6333>GFP* larvae (T-test, *P*>0.05, N = 10).
(TIF)

Figure S2 Mutation of *FOXO* attenuates the effects of loss of *Akt* on the rate cell proliferation in the eye-antennal and wing discs but not in the genital discs. Columns with the same letter are not significantly different (Tukey HSD, *P*>0.05). Error bars are 1 standard error.
(TIF)

Figure S3 Manipulating *FOXO* expression in the developing wing imaginal disc. Driving expression of *FOXO.RNAi* and *FOXO.wt* in the wing imaginal disc using *NP6333* (gray bars), results in a significant decrease or increase in *FOXO* expression respectively, compared to wild-type controls (open bars) (** T-test, *P*<0.01, *** T-test, *P*<0.001, N = 5). Error bars are 1 standard error.
(TIF)

Figure S4 Changes in *FOXO* expression in the wing imaginal discs have organ autonomous effects. (A) The scaling relationship between leg length and body size for wild-type (open diamonds) and *NP6333>FOXO.wt* (closed diamonds) flies reared at 23°C are not significantly different (common slope test, *p* = 0.725). (B) Final body size was the same for *NP6333>FOXO.wt* and *N6333>GFP* control flies reared at 23°C. Two-sample t-test, *p* = 0.175, N = 26 (>*FOXO.wt*), 28 (>*GFP*).
(TIF)

Figure S5 Altering *FOXO* expression using the temperature dependence of GAL4 activity. Rearing *NP6333>FOXO.wt* larvae at higher temperatures results in a significant increase in *FOXO* expression in the wing imaginal discs (linear regression, *p* = 0.0142).
(TIF)

Figure S6 The relationship between temperature, wing nutritional plasticity and *FOXO* expression in wild-type *Drosophila* (A) In wild-type larvae, an increase in rearing temperature leads to a decrease in *FOXO* expression in the wing imaginal discs (linear regression, *p*<0.001) (B) As temperature increases there is a significant trend towards decreased nutritional plasticity of the wing in wild-type flies (permutation test *p* = 0.033). The permutation procedure was to first transform the raw data so that the bivariate mean for the wing and body size at each temperature was zero. The transformed data for all rearing temperatures were pooled and re-sampled without replacement, to create four new permuted data sets, one for each temperature. The slope of the SMA for each of these datasets was then regressed against temperature, to generate a regression coefficient (*b_A_*). This was repeated 1000 times to produce a distribution of regression coefficients under the null hypothesis that there is no relationship between plasticity and temperature. The position of the observed regression coefficient (*b_O_* = −0.0105) was determined among the ordered coefficients (*b_A_*) from the permuted datasets. The proportion of *b_A_* less than or equal to *b_O_* was used as the *p*-value under the null hypothesis.
(TIF)
