# Supplementary figures and images for: FOXO Regulates Organ-Specific Phenotypic Plasticity In Drosophila
Source: PLoS Genet. 2011 Nov 10;7(11):e1002373. doi: 10.1371/journal.pgen.1002373 (PMC3213149; doi:10.1371/journal.pgen.1002373)

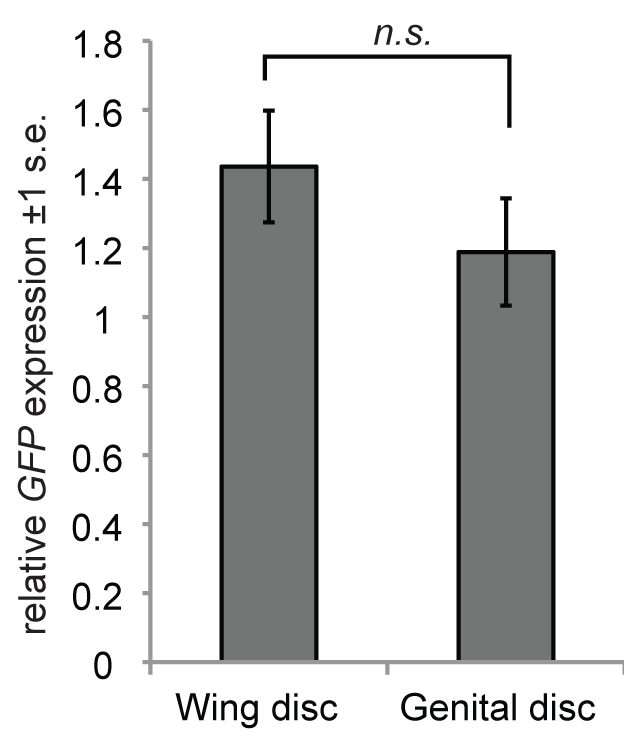

Supplement: Figure S1 — NP6333 drives expression equally in the wing and genital imaginal discs. Expression of GFP (assayed by qPCR) 39 hours after ecdysis from the second to the third larval instar is not significantly different in wing and genital discs of NP6333>GFP larvae (T-test, P>0.05, N = 10). (TIF) [file pgen.1002373.s001.tif]

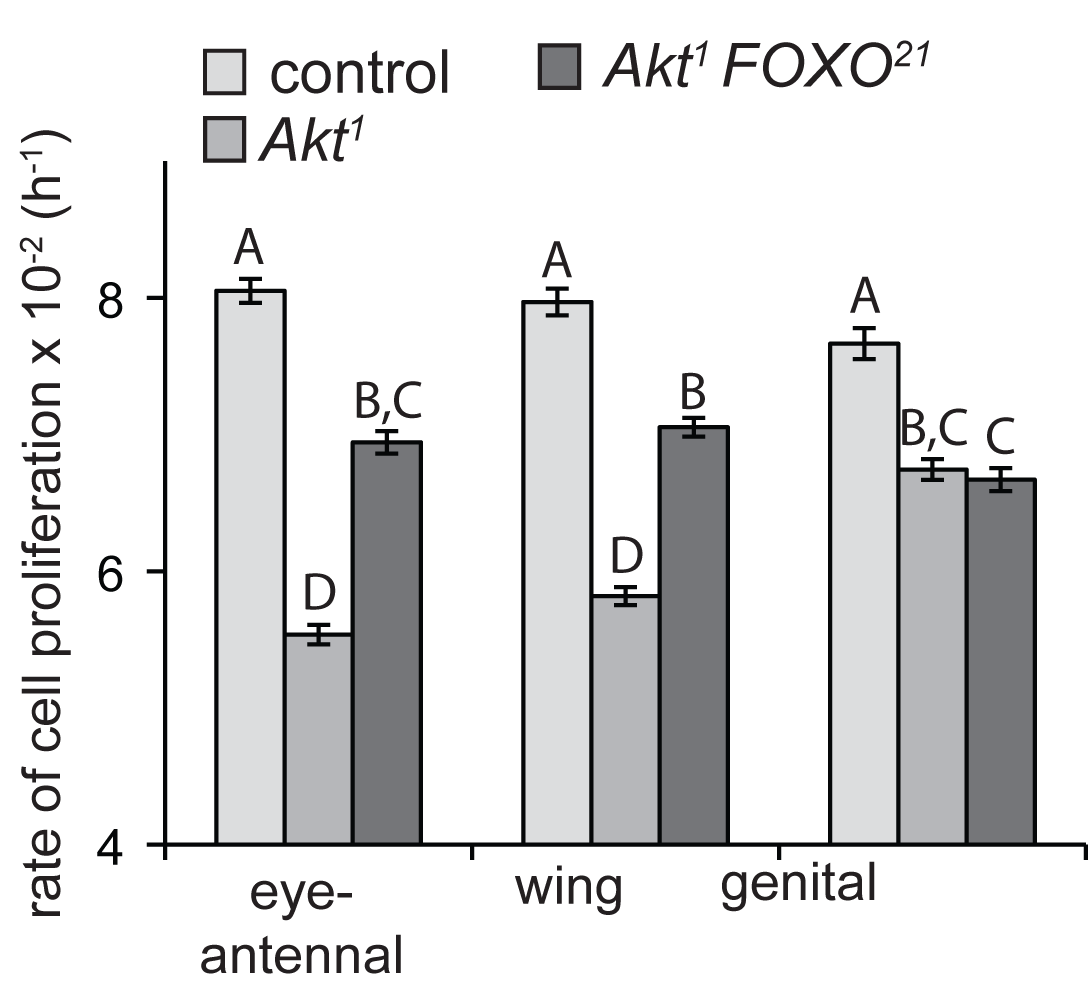

Supplement: Figure S2 — Mutation of FOXO attenuates the effects of loss of Akt on the rate cell proliferation in the eye-antennal and wing discs but not in the genital discs. Columns with the same letter are not significantly different (Tukey HSD, P>0.05). Error bars are 1 standard error. (TIF) [file pgen.1002373.s002.tif]

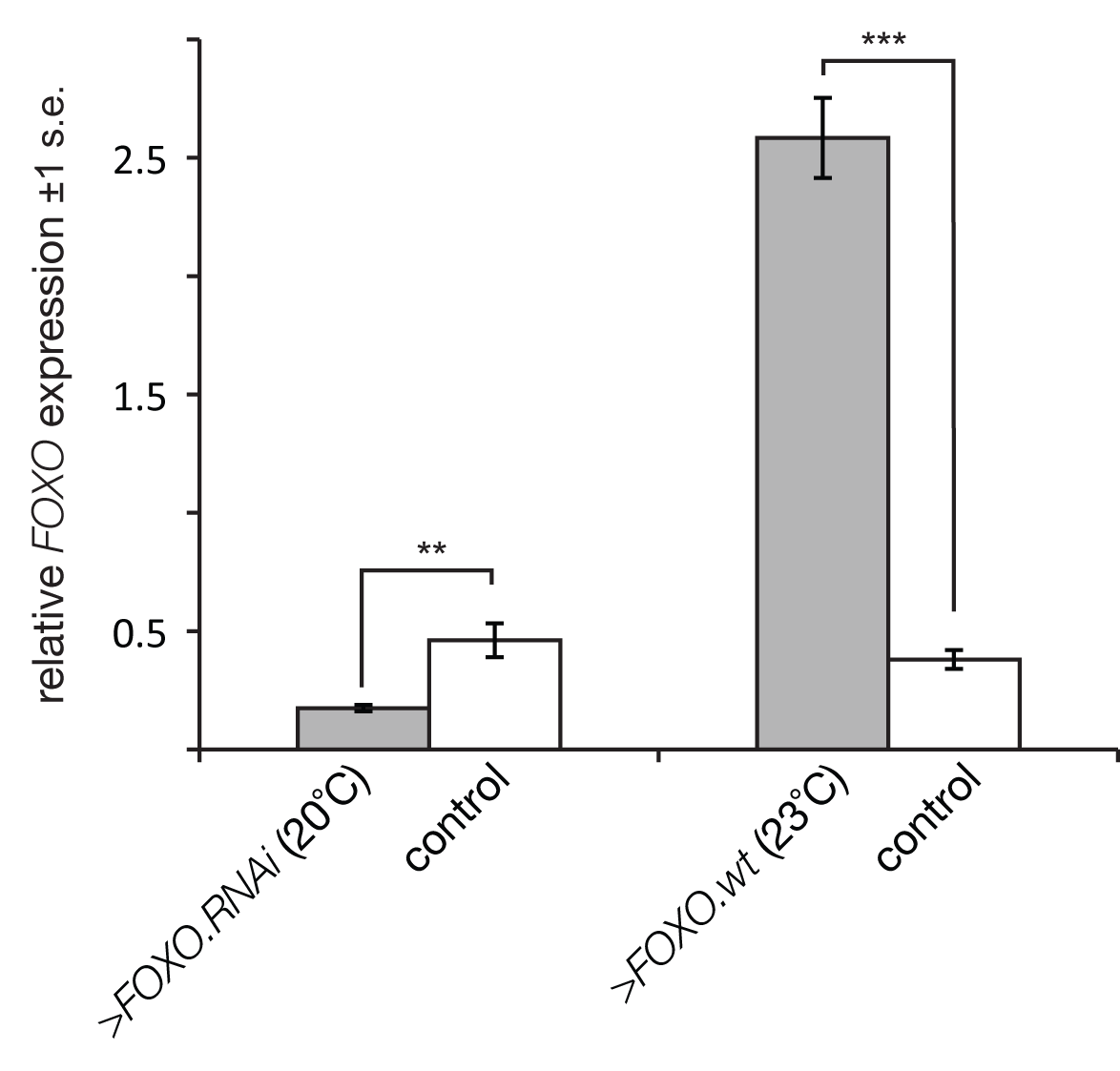

Supplement: Figure S3 — Manipulating FOXO expression in the developing wing imaginal disc. Driving expression of FOXO.RNAi and FOXO.wt in the wing imaginal disc using NP6333 (gray bars), results in a significant decrease or increase in FOXO expression respectively, compared to wild-type controls (open bars) (** T-test, P<0.01, *** T-test, P<0.001, N = 5). Error bars are 1 standard error. (TIF) [file pgen.1002373.s003.tif]

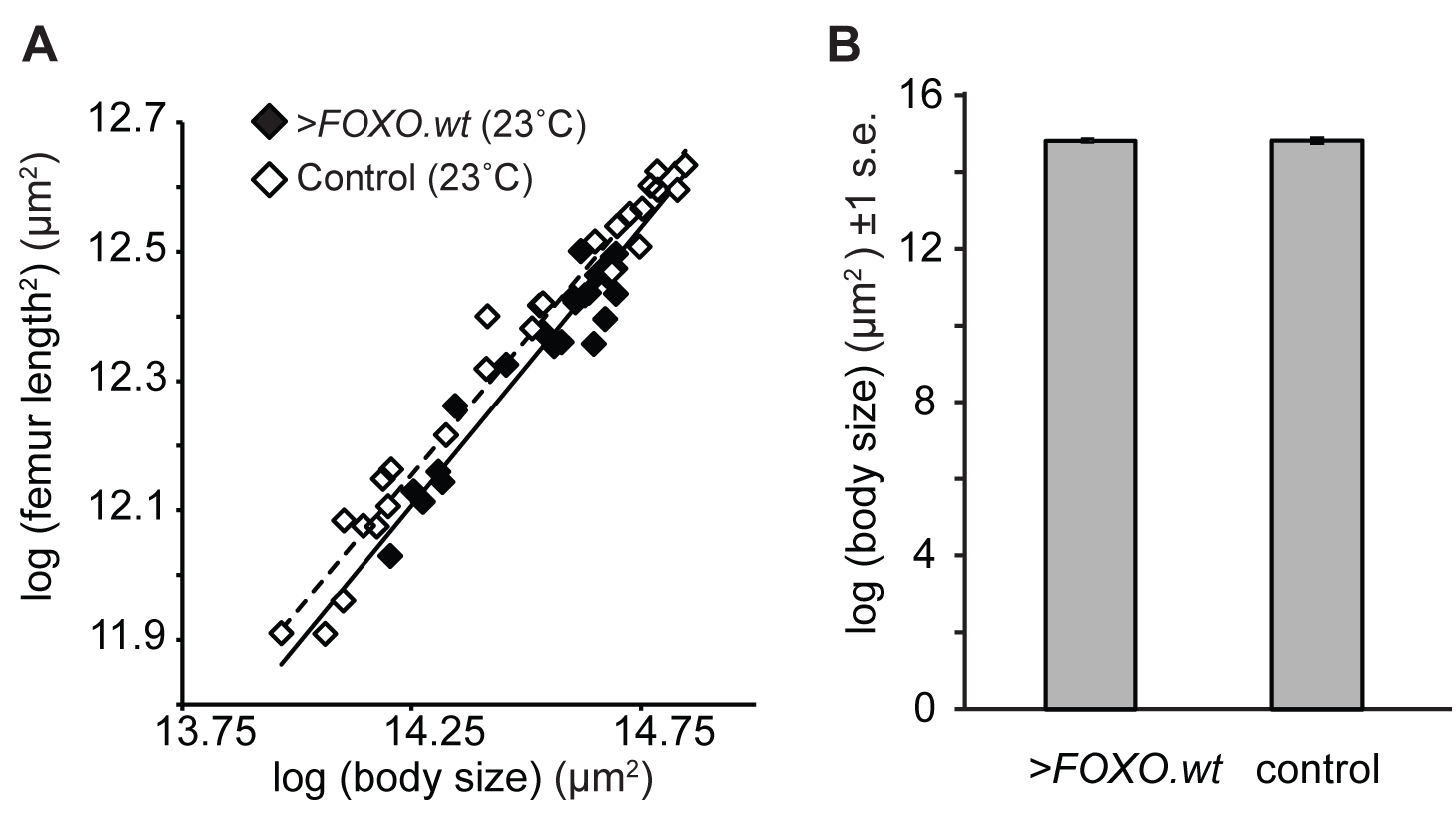

Supplement: Figure S4 — Changes in FOXO expression in the wing imaginal discs have organ autonomous effects. (A) The scaling relationship between leg length and body size for wild-type (open diamonds) and NP6333>FOXO.wt (closed diamonds) flies reared at 23°C are not significantly different (common slope test, p = 0.725). (B) Final body size was the same for NP6333>FOXO.wt and N6333>GFP control flies reared at 23°C. Two-sample t-test, p = 0.175, N = 26 (>FOXO.wt), 28 (>GFP). (TIF) [file pgen.1002373.s004.tif]

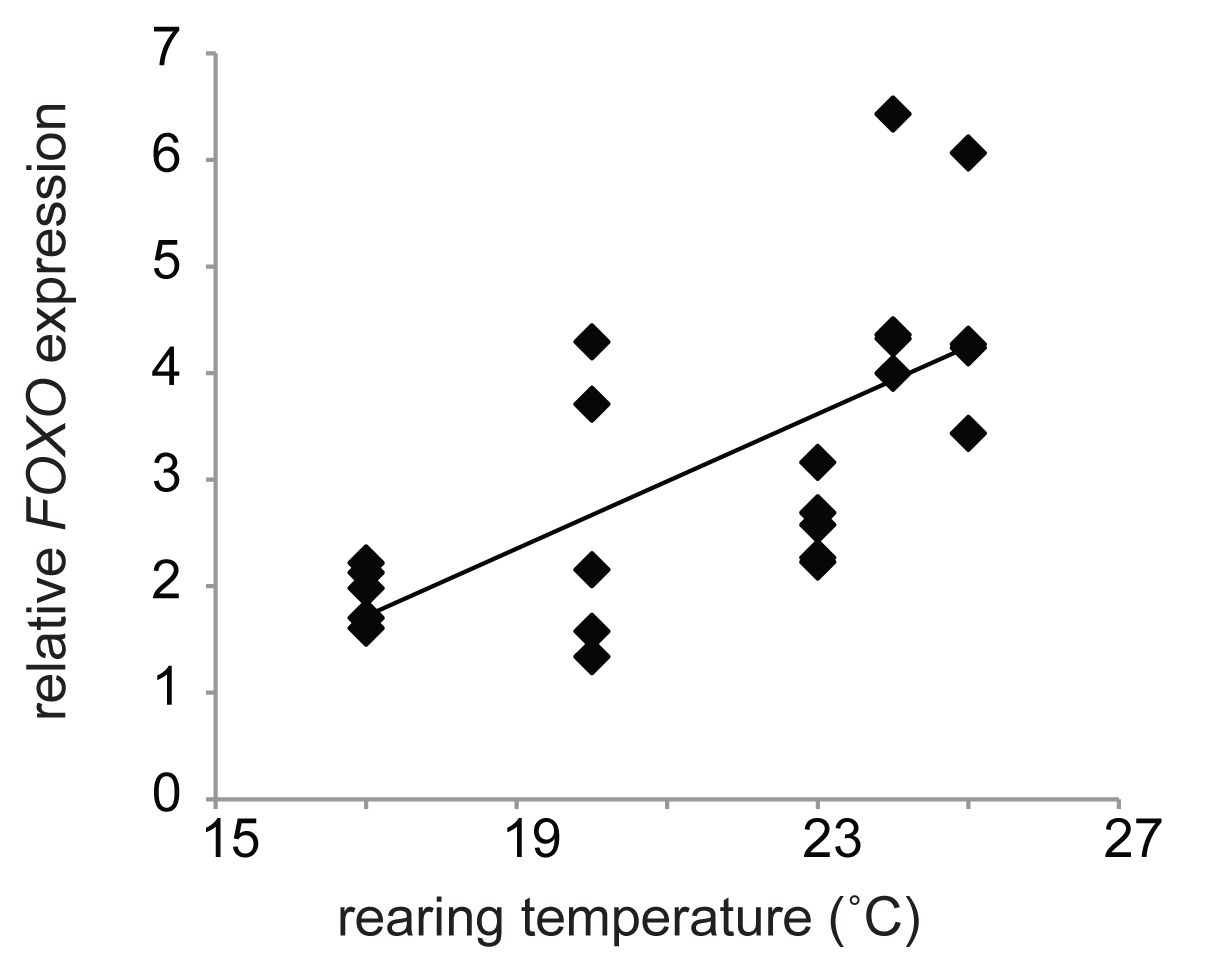

Supplement: Figure S5 — Altering FOXO expression using the temperature dependence of GAL4 activity. Rearing NP6333>FOXO.wt larvae at higher temperatures results in a significant increase in FOXO expression in the wing imaginal discs (linear regression, p = 0.0142). (TIF) [file pgen.1002373.s005.tif]

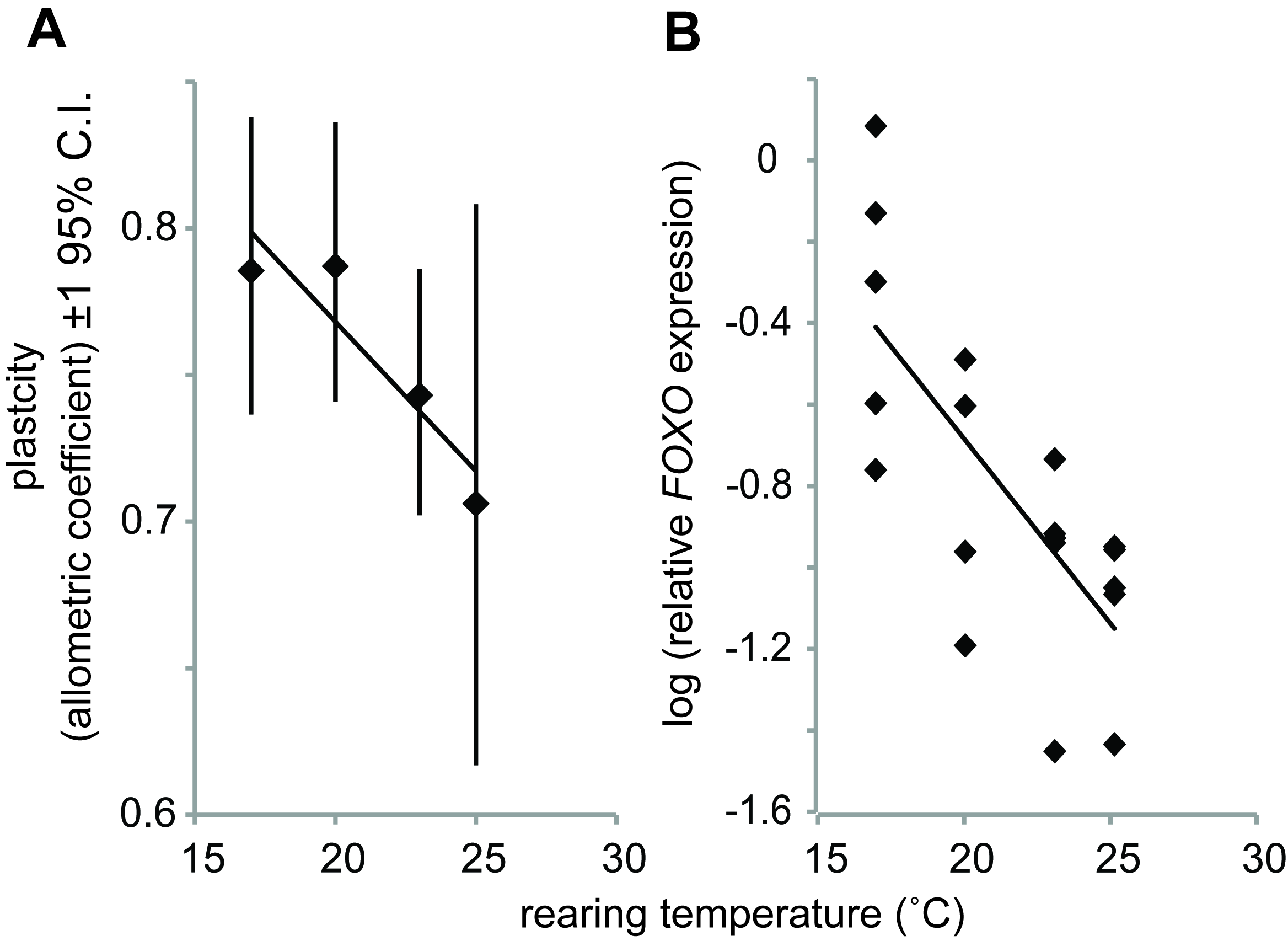

Supplement: Figure S6 — The relationship between temperature, wing nutritional plasticity and FOXO expression in wild-type Drosophila (A) In wild-type larvae, an increase in rearing temperature leads to a decrease in FOXO expression in the wing imaginal discs (linear regression, p<0.001) (B) As temperature increases there is a significant trend towards decreased nutritional plasticity of the wing in wild-type flies (permutation test p = 0.033). The permutation procedure was to first transform the raw data so that the bivariate mean for the wing and body size at each temperature was zero. The transformed data for all rearing temperatures were pooled and re-sampled without replacement, to create four new permuted data sets, one for each temperature. The slope of the SMA for each of these datasets was then regressed against temperature, to generate a regression coefficient (bA). This was repeated 1000 times to produce a distribution of regression coefficients under the null hypothesis that there is no relationship between plasticity and temperature. The position of the observed regression coefficient (bO = −0.0105) was determined among the ordered coefficients (bA) from the permuted datasets. The proportion of bA less than or equal to bO was used as the p-value under the null hypothesis. (TIF) [file pgen.1002373.s006.tif]
